# The method for impact analyzing of duplex plasma nitriding and DLC coatings on the cyclic performance of 16MnCr5 piston pin steel under plain and fretting fatigue testing

**DOI:** 10.1016/j.mex.2025.103164

**Published:** 2025-01-08

**Authors:** Mobin Dizisaz, Mohammad Sadegh Aghareb Parast, Mohammad Azadi

**Affiliations:** Faculty of Mechanical Engineering, Semnan University, Semnan, Iran

**Keywords:** Fretting fatigue, Plain fatigue, Coating, Diamond like carbon, Plasma nitriding, Piston pin, 16MnCr5 steel alloy, The Poisson method, and the linear regression (no transform) method

## Abstract

The current analysis studied the analyzing method and experimental datasets of the plain and fretting fatigue lifetime of 16MnCr5 steels and the impact of duplex plasma nitriding and diamond-like carbon (DLC) coatings. Standard specimens were cut from industrial piston pins used in combustion engines and machined for this objective. Subsequently, plain and fretting fatigue tests, with a fretting force of 15 N, were performed on both as-received and DLC-coated specimens at stress levels ranging from 250 to 550 MPa and under a frequency of 100 Hz. The impact of the mentioned parameters on the fatigue behavior of the samples was carefully analyzed using regression models to identify the influential or significant factors.

• Fully reversed rotary bending plain and fretting fatigue testing was conducted at stress levels ranging from 250 to 550 MPa. The fatigue lifetime of the coated samples increased by 47.7 % under pure fatigue conditions and by 85.3 % under fretting fatigue conditions, respectively.
• OM and Raman spectroscopy were used to investigate the microstructure and the DLC coating. The thickness of the DLC coating was measured at 1.956± 0.1478 micrometers, while the thickness of the white layer was 4.4248± 0.5020 micrometers.
• Obtained fatigue data were analyzed in Design-Expert software using the Poisson method and the linear regression (no transform) method. In the results of the Poisson regression model for all fatigue data, the McFadden Pseudo R² was 94.92 %, and the Adjusted McFadden Pseudo R² was also 94.92 %. For the linear regression model analyzing all logarithmic fatigue data, the R² was 92.87 %, the Adjusted R² was 90.75 %, and the Predicted R² was 82.76 %.

## Graphical abstract

**Figure:**
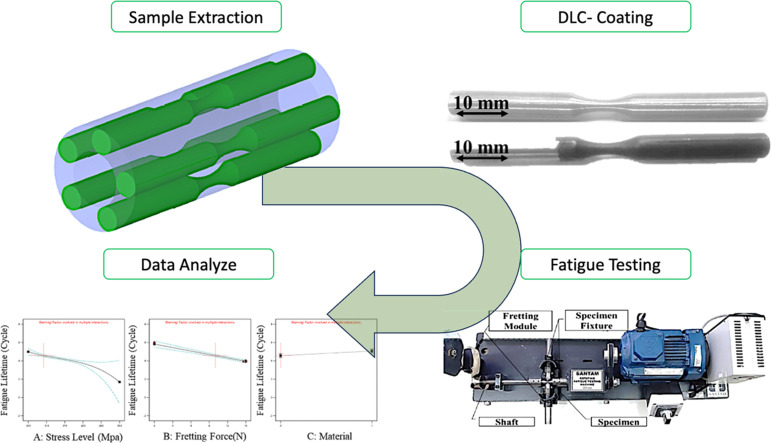

Specifications table

**Table:** 

Subject area:	Engineering
More specific subject area:	Engineering/ Manufacturing Engineering/ Mechanical Engineering/ Automotive Engineering
Name of your method:	The Poisson method, and the linear regression (no transform) method
Name and reference of original method:	M. Dizisaz, M. S. Aghareb Parast, M. Azadi and A. Dadashi, Plain and fretting fatigue characteristics of 16MnCr5 steel under cyclic bending loading with the application of engine piston pin, Materials Chemistry and Physics, Vol. 316, Article No 129,110, 2024.
Resource availability:	Repository name: Mendeley Data 10.17632/564gdrxv79.1 Direct link to the dataset: https://data.mendeley.com/datasets/564gdrxv79/1 Azadi, Mohammad; Dizisaz, Mobin; Aghareb Parast, Mohammad Sadegh (2024), “HCF testing raw data on piston pin steels”, Mendeley Data, V1

## Background

This dataset was compiled to advance our understanding of the fatigue phenomena in 16MnCr5 steels, particularly under plain and fretting fatigue conditions, and to evaluate the effectiveness of duplex plasma nitriding and diamond-like carbon coatings. The standard specimens, representative of piston pins in combustion engines, were subjected to fatigue testing at varying stress levels and frequencies to simulate actual conditions.

16MnCr5 steel is used to make durable machine parts like gears and shafts in engines [1]. Fatigue and fretting fatigue can cause cracks initiation in piston pins due to cyclic loading and high temperatures [2]. Understanding the fretting and fatigue behaviors of materials used in piston pins is essential for ensuring engine reliability and durability. Fatigue damage can generally be prevented or minimized by enhancing material selection, manufacturing processes, and maintenance of the piston pin. Additionally, various coating methods, such as diamond-like carbon (DLC) coatings, are widely used in engine parts. These coatings offer potential benefits, including improved fuel efficiency, reduced carbon emissions, and extended service life [3]. Designers in automotive engineering can focus on applying DLC coatings to engine parts due to their excellent chemical stability, low friction, seizure resistance, and high hardness. [4]. DLC coatings cover surface defects of the host material, enhancing its fatigue and mechanical properties [5]. Bartels et al. [6] examine the in-situ modification of case-hardened 16MnCr5 using laser metal deposition. This article presents an experimental analysis of the microstructure and hardness of 16MnCr5 modified with carbon and tungsten carbide using laser metal deposition. It discusses the impact of various processing parameters and alloying elements on the phase formation and wear resistance of 16MnCr5. Moreover, Berger et al. [7] studied the rolling contact fatigue of coated 16MnCr5 steel substrates. The results showed that the durability of the coatings could be enhanced by using a quenched and tempered substrate. Based on studies [8], fretting damage can reduce the fatigue life of DLC-coated 16MnCr5 by causing cracks and delamination in both the coatings and the substrate. This damage can be mitigated by optimizing the design of steel parts, material selection, coating process, and operating conditions. Excellent adhesion is crucial in coatings technology. However, achieving high adhesion for DLC coatings in automotive engines is challenging due to differences in physical properties, such as thermal expansion coefficient, elastic modulus, hardness, and yield/ultimate strength between the substrate and coating layers [9].

This dataset offers insights into the factors that significantly influence material fatigue endurance. The regression models employed in the analysis highlight the dataset's potential to inform better predictive models and engineering practices, thereby adding substantial value to the foundational research. This study, considering the significance of investigating fatigue and fretting fatigue in the mentioned alloy, used regression models to analyze the fatigue data. This approach helps identify how factors such as fretting damage and DLC coating influence fatigue lifetime under various stress levels.

## Method details

The study investigated the lifetimes of 16MnCr5 steel under both plain and fretting fatigue conditions. It also explored the impact of duplex plasma nitriding and diamond-like carbon (DLC) coatings on this steel. The fatigue behavior of the samples was analyzed using regression models to identify significant influencing factors.

Two methods were employed: the Poisson method for analyzing all fatigue lifetime data and linear regression (without transformation) to study the fatigue lifetime on a logarithmic scale. The inputs included stress level, fretting force, and material, while the responses were the fatigue lifetime and its logarithmic values.

**Fig. 1:**
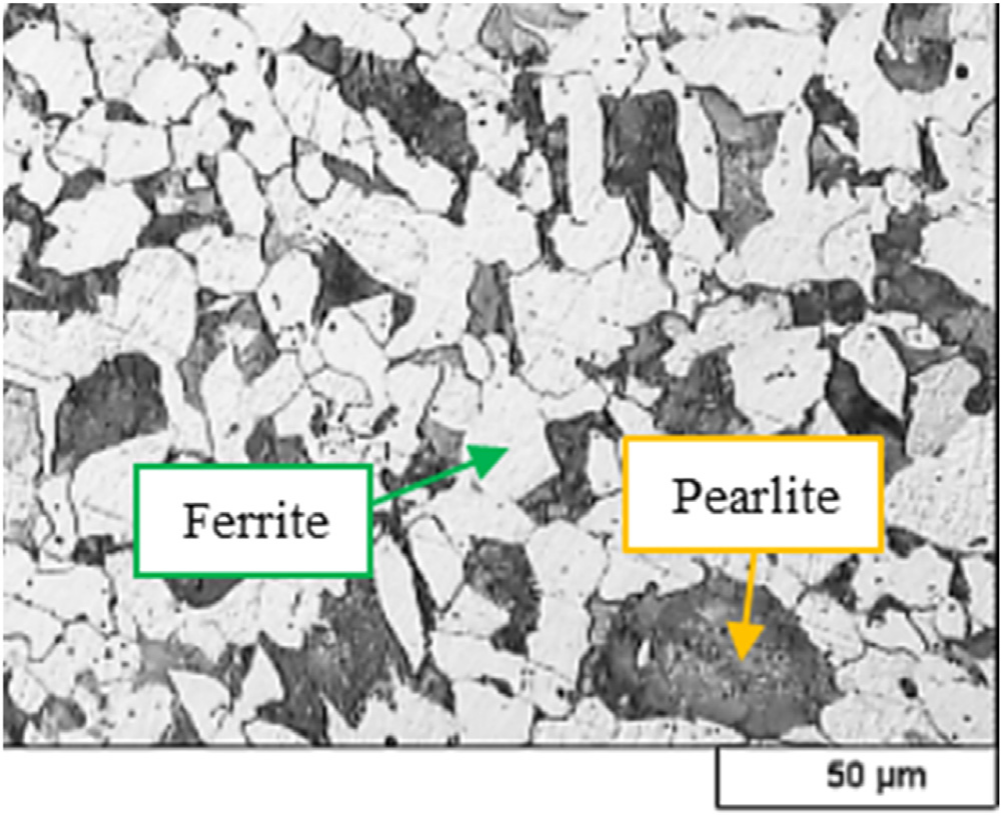
Microstructures of the studied sample with 200X magnification.

The investigated material was piston pin steel (16MnCr5) extracted from the industrial piston pins ICE used in a passenger car engine. Using quantometry testing according to the ASTM-A751/ASTM-E415 standards [10,11], the chemical elements in the material composition are reported and represented in Table 1. Moreover, compared to the data of the DIN standard [12], it was proved that the used material was 1.7131 steel (DIN-16MnCr5). The microstructure of the studied sample is reported in Fig. 1. To examine the microstructure, the sample was polished and then etched with a 2 % Nital solution (containing 2 ml of nitric acid and 100 ml of ethanol) for about 5 s to investigate the different phases of the alloy.

**Table 1:** The chemical composition (wt.%) for 16MnCr5 steel alloy.

Reported Material	C	Si	Mn	P	S	Cr	Ni	Al	Co	Cu	Fe
Studied Material	0.18	0.26	1.10	0.014	0.007	0.91	0.03	0.016	0.01	0.01	Base
Minimum value*	0.14	0	1.00	0	0	0.80	–	–	–	–	Base
Maximum value*	0.19	0.40	1.30	0.025	0.035	1.10	–	–	–	–	Base

⁎ Based on Standard EN 10,084:2008 for 16MnCr5 [12].

It should be noted that no surface pre-treatments, such as grinding or polishing, were applied to the samples. Additionally, although surface roughness can influence fatigue behavior, it was not measured in this study. This is because fatigue cracks can initiate from the surface of the samples and propagate towards the center [13]. Various approaches, such as heat treatment and DLC coating, can reduce wear in samples, minimize friction, dissipate heat, provide surface protection, and remove contaminants in the case of lubricants. These methods can significantly enhance the durability and longevity of piston pins in multiple applications [14,15]. To improve the fatigue property of the piston pin, the samples were coated by DLC. The performance of DLC coating depends on the strength of the adhesion between the substrate and coatings. However, DLC coatings cannot be directly fabricated on steel since the materials cannot attach firmly to the substrate. Therefore, the substrate requires third-party assistance, an intermediate layer promoting adhesion. DLC can be applied to the top of the middle layer. The intermediate layer should be chosen to bond strongly to the substrate and the coating [14]. It could be noted that both DLC coating methods were plasma-assisted chemical vapor deposition (PACVD), which is an effective technique for DLC coating deposition that is a significant factor that affects DLC coating properties and is widely used for automotive applications [16].

Plasma-Assisted Chemical Vapor Deposition (PACVD) is a technique for depositing thin films, including diamond-like carbon (DLC) coatings, onto various substrates. The process involves using a plasma, often generated by mid-frequency pulsed bias, to crack precursor gases like acetylene (C_2_H_2_) at relatively low temperatures. The plasma nitriding plus DLC coating parameters are shown in Table 2. Initially, to reduce residual stress and enhance DLC coating adhesion on the steel surface, the samples were plasma-nitrided and exposed to a hydrogen and nitrogen gas mixture at a 3:1 ratio under 3.5 millibars of pressure. Subsequently, the DLC coating process was carried out with a voltage of 500 V, a current of 3 A, and a frequency of 15 kHz. The samples were plasma nitrided and DLC-coated by Plasma Pazhouh Pars Company, which used its custom-made device. Diamond-like carbon (DLC) coatings in this study consisted of an amorphous network of sp^2^ and sp^3^ C—C bonds and hybrid C—H bonds. Different types can be mentioned as follows: Ta-C: Tetrahedrally bound hydrogen-free amorphous carbon, known for its high hardness, a-C:H: Amorphous carbon with hydrogen, which affects its mechanical properties and stability, and Ta-C:H: Tetrahedrally bound amorphous carbon with hydrogen. Each type has different proportions of sp^3^ (diamond) and sp^2^ (graphite) bonds, affecting the coating hardness, friction, and wear resistance. The DLC coating is applied on the surface of the sample, covering the whole middle part of the test specimen.

**Table 2:** Coating parameters in this study [16].

Parameters	Plasma nitriding				DLC coating			
	Temperature	Time	Voltage	Duty cycle	Temperature	Time	Pressure	Gases ratio
	(°C or K)	(hr.)	(V)	(on/off time)	(°C or K)	(hr.)	(Pa)	–
Values	450 °C or 723.15 K	2	450	0.65	200 °C or 473.15 K	2	200	Ar:75 % CH_4_:25 %

**Fig. 2:**
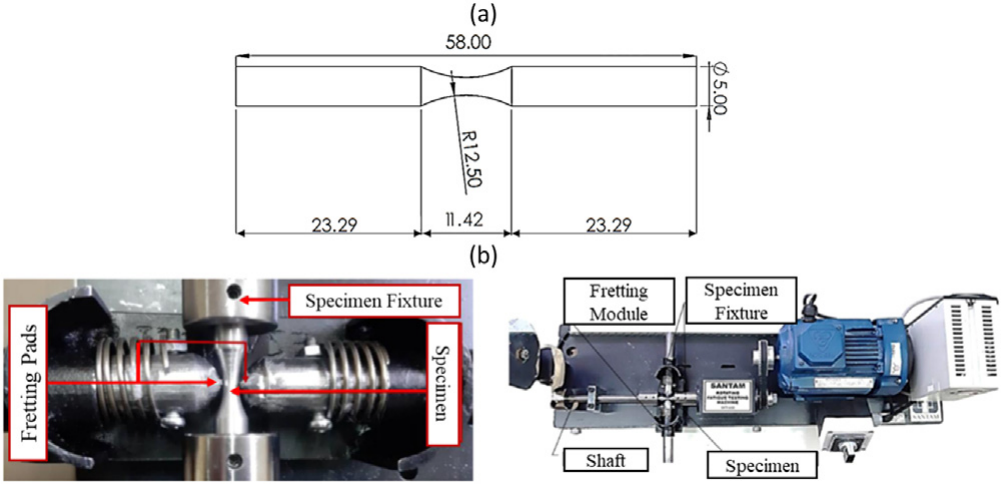
(a) The standard fatigue testing sample geometries and (b) the fretting module and the fatigue testing machine [20].

**Fig. 3:**
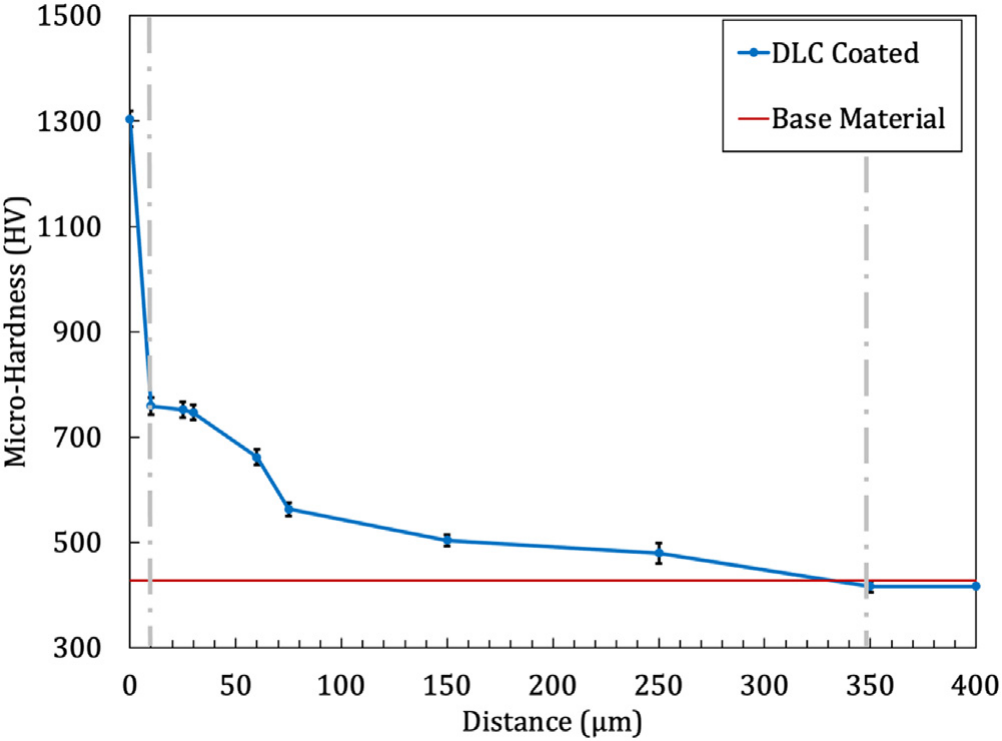
The hardness of as-cast and DLC-coated specimens.

It must be noted that the geography of the specimen and fatigue test condition were considered based on DIN-EN-50,113 and ISO-1143 standards [17,18]. The fatigue test device was Santam-SFT-600 (two points/rotary bending loading). In addition, a fretting module was used to apply the fretting condition, which inserts constant fretting normal load on the surface of the sample using springs. The fretting loading and the testing frequency were considered 15 N [19] and 100 Hz (rated-power condition of automobile engines [20]). The sample geometries and fatigue testing machine are shown in Fig. 2. The fatigue tests were performed at 250 to 550 MPa stress levels to evaluate the rotating bending fatigue behavior of the material. It should be noted that all PF and FF experiments were done at room temperature. Further details about the fatigue test machine, the used fretting module, and the fretting pad material can be found in the previous works of the author [21]. Vickers hardness of the as-received sample was measured using the Innova-test Nova 240 device, and changes after DLC coating were examined with the Micro Vickers hardness tester MMT-X, following ASTM E-384 standards [22]. The hardness measurement was conducted using an applied load of HV0.3 (2.942 N) for a duration of 10–15 s. The hardness of the as-received samples was 428± 2.94 Vickers (approximately 43 Rockwell Hardness (HRC) or 400 Brinell Hardness (HB)). Literature [23] reports that the hardness of piston pins varies with tempering temperatures between 250 and 500 °C (523.15 to 773.15 K), resulting in hardness values from 400 to 600 Vickers. Fig. 3 compares the microhardness contours of coated samples with as-received samples, highlighting three main areas: the coated layer, diffusion zone, and core sample. The hardness of the coated surface in DLC specimens was about 1303.85± 14.91 Vickers (approximately 92 HRC or 1300 HB). Hardness values gradually decrease towards the core of the coated samples, stabilizing around 375 µm from the surface, indicating the Nitrogen diffusion zone.

**Fig. 4:**
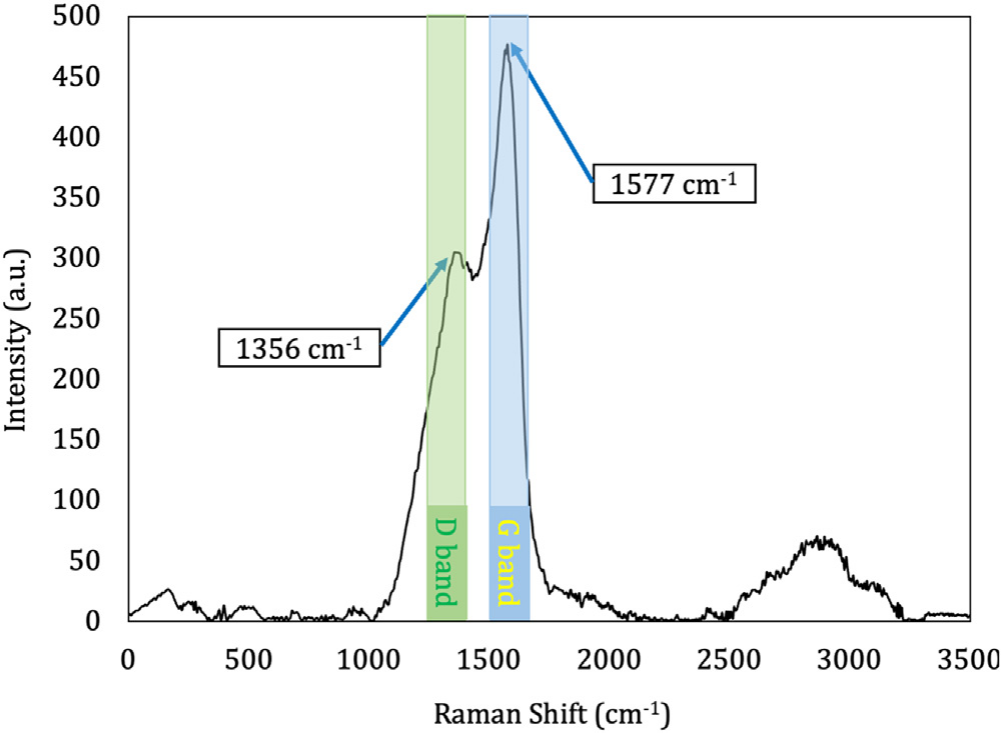
Raman spectra of the coated sample.

Utilizing the Raman Takram P50C0R10 instrument, the formation of a pseudo-diamond carbon layer was assessed. This evaluation employed a 532 nm wavelength, a laser output of 150 mW, and a resolution of 6 cm^-1^. The chemical impact of DLC coatings on sample surfaces was examined through Raman spectroscopy, with findings illustrated in Fig. 4. The Raman spectral analysis of the DLC films revealed two distinct, overlapping bands, the G and D bands. The synthesis process of the coating is reflected in the Raman spectrum, which displays two characteristic peaks: the D peak, indicative of Diamond, and the G peak, associated with Graphite. The D peaks representing sp^3^ amorphous carbon were observed at approximately 1350–1370 cm^-1^, consistent with other literature references. The G band peaks were noted at around 1570–1580 cm^-1^. The intensity ratio of the peaks (ID/IG) for DLC coatings showed values of approximately 0.7. These G and D peaks were identified from the raw spectrum using peak fitting techniques. The characteristics of the D and G bands, such as their properties, were then employed to deduce alterations in the bonding structure within the amorphous DLC coatings. Within these coatings, the width of the G peak inversely correlates with the quantity and size of sp^2^ clusters, while the position of the G peak shifts higher with an increasing sp^2^/sp^3^ bonding ratio. Based on these peak observations, it can be inferred that DLC coatings were effectively applied to the sample surfaces [16].

## Method validation

The present study examined experimental data about the lifetimes of 16MnCr5 steel under plain and fretting fatigue conditions. Additionally, it investigated the effects of duplex plasma nitriding and diamond-like carbon (DLC) coatings on this steel. The fatigue behavior of the samples was analyzed using regression models, which facilitated the identification of the significant factors influencing the outcomes.

Two methods were used: first, all the fatigue lifetime data was analyzed using the Poisson method, and second, the logarithm of the obtained fatigue lifetime was analyzed using the linear regression (no transform) method. Two methods were used to analyze the fatigue lifetime data. First, the Poisson method was employed. This statistical method is beneficial for modeling count data and rare events. It assumes that the number of events occurring within a fixed interval of time or space follows a Poisson distribution characterized by a mean rate of occurrence. In the context of fatigue lifetime analysis, the Poisson method helps understand the distribution and frequency of fatigue failures over time. Second, the logarithm of the obtained fatigue lifetime was analyzed using the linear regression method without transformation. Linear regression is a fundamental statistical technique used to model the relationship between a dependent variable and one or more independent variables. By taking the logarithm of the fatigue lifetime, we aim to linearize the relationship, making it easier to apply linear regression. This approach allows us to identify trends and make predictions about fatigue lifetime based on the observed data. The inputs were stress level, fretting force, and material, as shown in Table 3, and the responses were the fatigue lifetime and the logarithm values of the fatigue lifetime. The ranges and details of these variables are defined in Table 4.

**Table 3:** The input definition in this study.

Factor	Name	Units Type	Minimum	Maximum	Mean	Std. Dev.
A	Stress Level (MPa)	Numeric- Continuous	250.00	550.00	390.82	77.51
B	Fretting Force (N)	Numeric- Continuous	0.00	15.00	8.27	7.54
C	Material	Categoric- Nominal	0 (As-received)	1 (DLC-coated)	–	–

**Table 4:** The description of the responses in this study.

Response	Name	Minimum	Maximum	Mean	Std. Dev.	Ratio
R1	Fatigue Lifetime (cycle)	3200	1.5617E+06	1.028E+05	2.771E+05	488.03
R2	Log Fatigue Lifetime	3.505	6.194	4.32	0.6621	1.77

**Fig. 5:**
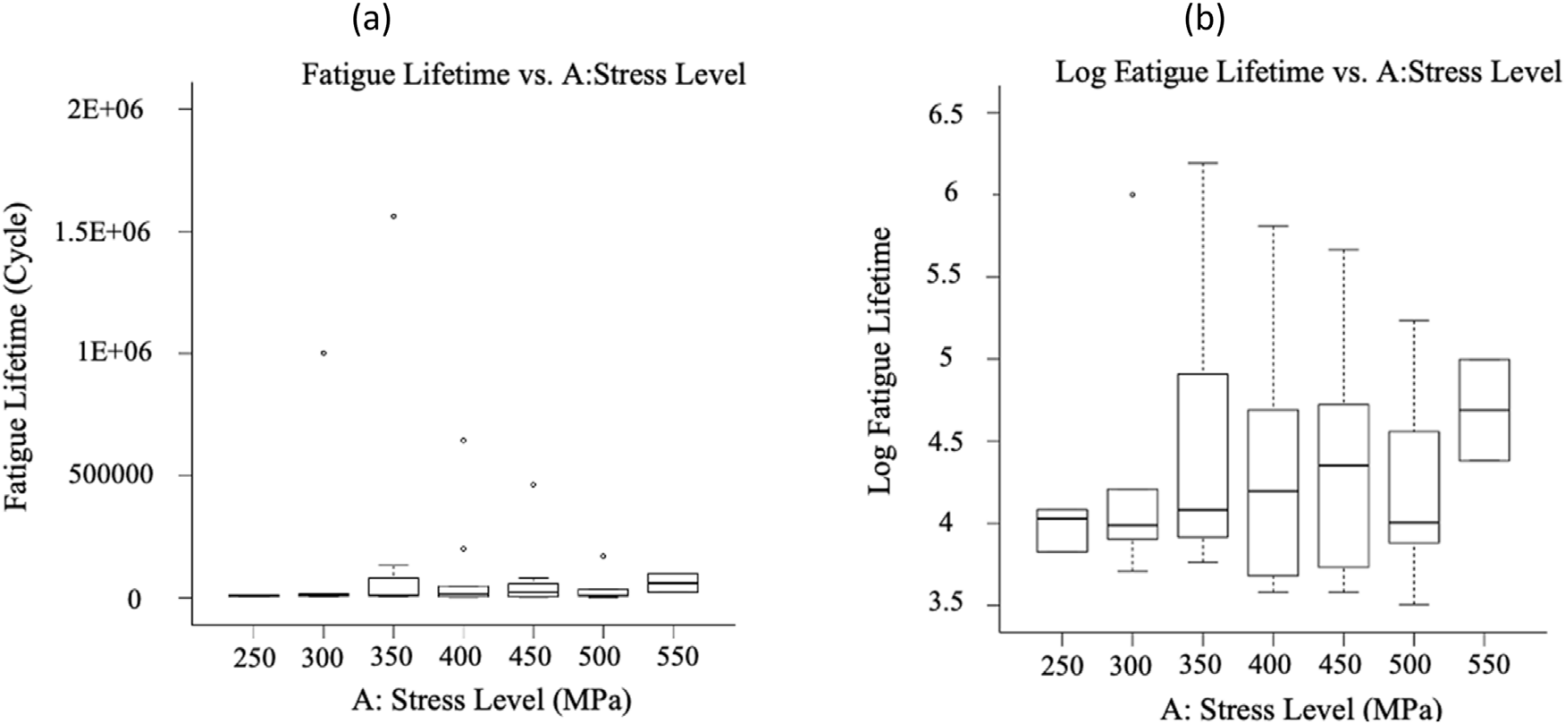
The variation of the responses regarding the stress level, including (a) the fatigue lifetime and (b) the log fatigue lifetime.

**Fig. 6:**
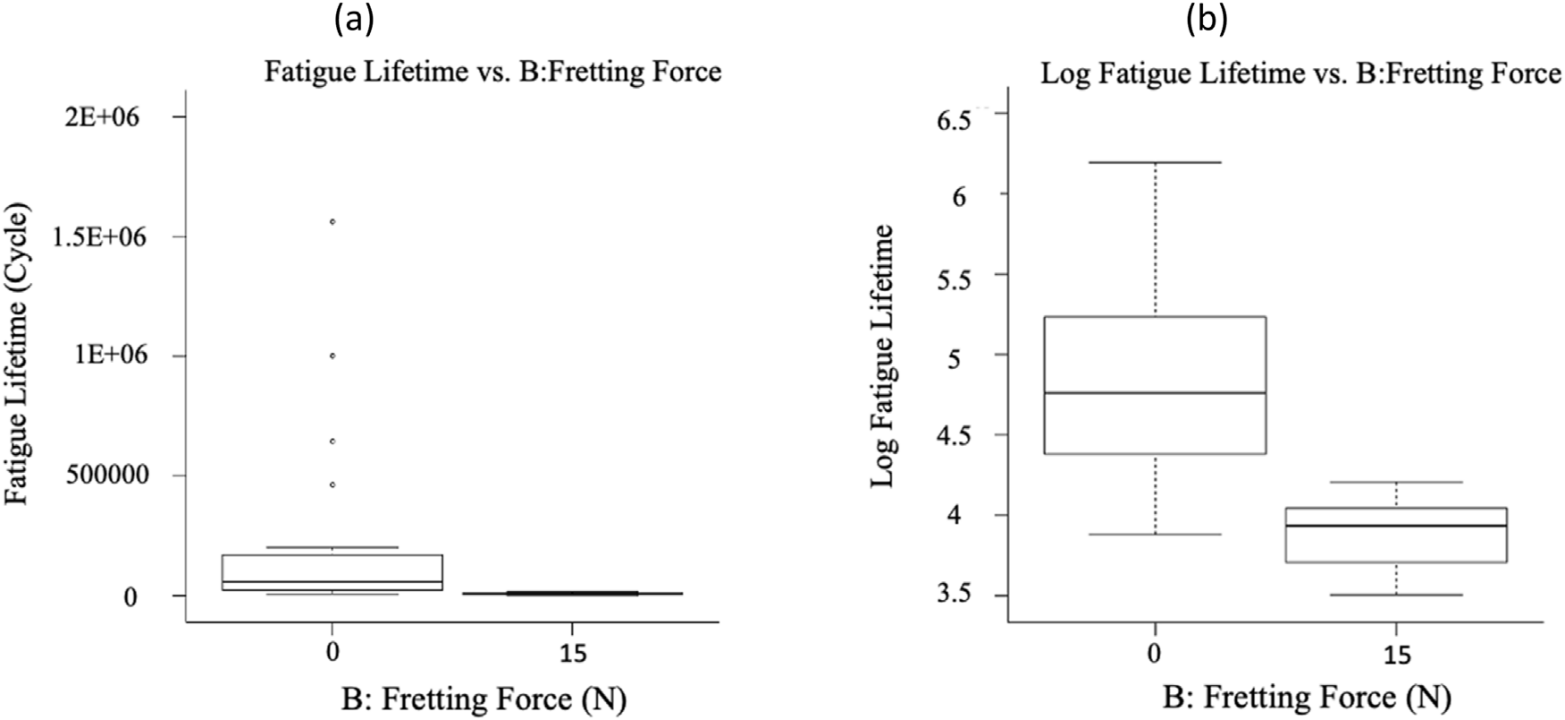
The variation of the responses regarding the fretting force, including (a) the fatigue lifetime and (b) the log fatigue lifetime.

**Fig. 7:**
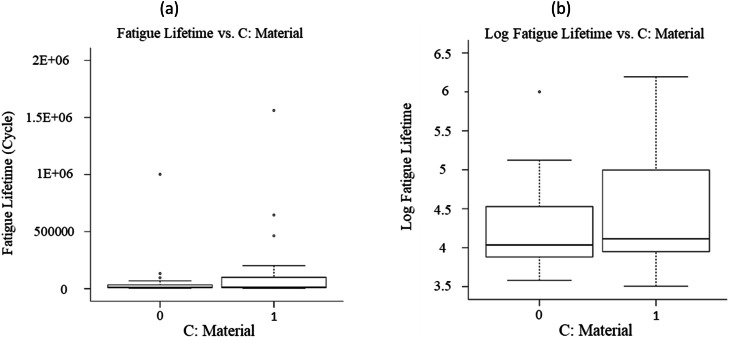
The variation of the responses regarding the material, including (a) the fatigue lifetime and (b) the log fatigue lifetime.

The fatigue datasets were initially analyzed by plotting all obtained data in Fig. 5, Fig. 6, Fig. 7, based on each factor, to visualize the scatter band and variation of each factor. This qualitative analysis revealed that stress level, fretting force, and applied coating significantly influenced the fatigue lifetime in 16MnCr5 steel alloys. It was observed that the fatigue lifetime varied noticeably across these three variables. Generally, fatigue lifetime is longer at lower stresses. However, the fatigue lifetime was lower at 250 and 300 MPa stress levels. This was because the experimental data at these stress levels included only fretting fatigue tests (see Fig. 1), whereas, at other stress levels, both plain fatigue examinations and fretting fatigue experiments were carried out. The reduction of the fatigue lifetime by inserting the fretting force is significant in Fig. 3. Fig. 4 presents the same issue for the material variation.

The fretting phenomenon can increase the shear and tensile stresses on the contact surface of samples, leading to the premature formation of cracks. This ultimately reduces the fatigue properties of the material and, ultimately, failure [24]. In addition, this decrease in strength can also be due to the presence of the stress concentration on the contact surface of specimens, containing the compressive contact loads and tangential stresses at the contact edge [25]. On the contact surfaces of samples, the fretting damage causes micro-crack formation, and then the crack growth occurs during cyclic loadings. These micro-cracks propagate in the subsurface of specimens. Thus, the fatigue lifetime can decrease according to the enhancement of the crack growth rate [26,27]. Fretting would expand the tensile and shear stresses on the contacting surfaces and create imperfections, which could cause early crack formation and, eventually, a decrease in the fatigue endurance of the samples [28].

Additionally, the cyclic stresses due to the engine combustion (pressure) that bend and deform the piston pin contacting surface emphasize the importance of studying the fretting effect on the fatigue behaviors of piston pin materials [29]. The obtained results indicate that the DLC-coated specimens show an improvement in fatigue lifetime. DLC coatings enhance fatigue lifetime through several mechanisms. They reduce friction, minimizing wear and damage from fretting. The DLC hardness and wear resistance protect against wear and damage, especially in high-stress, high-cycle fatigue conditions. The smooth surface finish reduces stress concentrations, improving fatigue life. The DLC chemical inertness shields against chemical degradation that can lead to fatigue failure. Additionally, certain DLC types can serve as solid lubricants, further reducing friction and wear, thus extending fatigue life [[30], [31], [32], [33]]. Hybridization states: sp^3^, sp^2^, and sp^1^. The DLC coating layers enable the film formation with various ratios between sp^3^ and sp^2^ hybridizations integrated into an amorphous structure. The properties of the material are more similar to those of diamond when the proportion of sp^3^ bonds is higher and its hardness and wear resistance increase. Conversely, the properties are more similar to those of graphite when the content of sp^2^ bonds is higher. Based on the structures, DLC coatings comprise an amorphous or disordered network in diamond with a short-range-ordered condition and the graphitic phases, distinguished by the sp^3^ tetrahedral and sp^2^ trigonal bonds, respectively [16].

Tables 5 and 6 display the results of the regression models for the fatigue lifetime and the logarithm values of the fatigue lifetime. In the Design-Expert software, the quantitative analysis indicates the proper regression model. This significance is supported by the small P-values and high coefficient of determination (R^2^) values exceeding 90 %, as reported in the tables. The adjusted R-squared is a modified version of R-squared that adjusts for predictors that are not significant in a regression model. Compared to a model with additional input variables, a lower adjusted R-squared indicates that the additional input variables are not adding value to the model. The regression model for all fatigue lifetime and the logarithmic scale of all values for the fatigue lifetime is depicted according to various inputs in Eqs. (1) and (2) as follows:

(1) $$\begin{array}{ccc} N_{Fatigue\;Lifetime} & = & 10.37-1.86A-1.38B+0.81C+0.52AB+0.20AC-0.43BC-0.39A^{2}\\ & & +\;0.38ABC-3.09A^{2}B+0.21A^{2}C-2.48A^{3} \end{array}$$

(2) $$\begin{array}{ccc} LogN_{Fatigue\;Lifetime} & = & 4.46-0.95A-0.63B+0.33C+0.32AB+0.10AC-0.15BC-0.05A^{2}\\ & & +\;0.29ABC-0.99A^{2}B+0.24A^{2}C-0.61A^{3} \end{array}$$

**Table 5:** The results of the Poisson regression model for all fatigue data.

Source	DF*	X2	P-value
Model	11	1.463E+07	< 0.0001
A-Stress Level	1	76,464.87	< 0.0001
B-Fretting Force	1	1.111E+06	< 0.0001
C-Material	1	1.895E+05	< 0.0001
AB	1	4522.13	< 0.0001
AC	1	1664.59	< 0.0001
BC	1	33,404.25	< 0.0001
A²	1	1569.97	< 0.0001
ABC	1	5733.91	< 0.0001
A²B	1	91,410.73	< 0.0001
A²C	1	1280.57	< 0.0001
A³	1	68,289.62	< 0.0001
Mean= 1.028E+05	–	McFadden Pseudo R²= 94.92 %	Adj. McFadden Pseudo R²= 94.92 %

⁎ DF: Degree of Freedom.

**Table 6:** The results of the linear regression model for all logarithmic fatigue data.

Source	SS*	DF	MS*	F-value	P-value	Effect
Model	19.540	11	1.780	43.800	< 0.0001	Significant
A-Stress Level	1.420	1	1.420	35.080	< 0.0001	Significant
B-Fretting Force	7.320	1	7.320	180.410	< 0.0001	Significant
C-Material	2.140	1	2.140	52.790	< 0.0001	Significant
AB	0.256	1	0.256	6.310	0.016	Significant
AC	0.039	1	0.039	0.958	0.334	Significant
BC	0.428	1	0.428	10.560	0.002	Significant
A²	0.003	1	0.003	0.079	0.780	Significant
ABC	0.078	1	0.078	1.940	0.172	Significant
A²B	0.264	1	0.265	6.530	0.0150	Significant
A²C	0.020	1	0.020	0.489	0.489	Significant
A³	0.060	1	0.059	1.460	0.234	Significant
Residual	1.500	37	0.041	–	–	–
Lack of Fit	0.241	7	0.034	0.8201	0.5783	Not Significant
Pure Error	1.260	30	0.0420	–	Std. Dev.= 0.2014	Adeq. Precision= 29.16
Cor Total	21.040	48	R2= 92.87 %	Adj. R2 = 90.75 %	Pre. R2 = 82.76 %	Mean = 4.32

⁎ SS: Sum of Squares; MS: Mean Squares.

As depicted in Table 3, *A* is the stress level, *B* is the fretting force, and *C* is the material (as-received and DLC-coated).

**Fig. 8:**
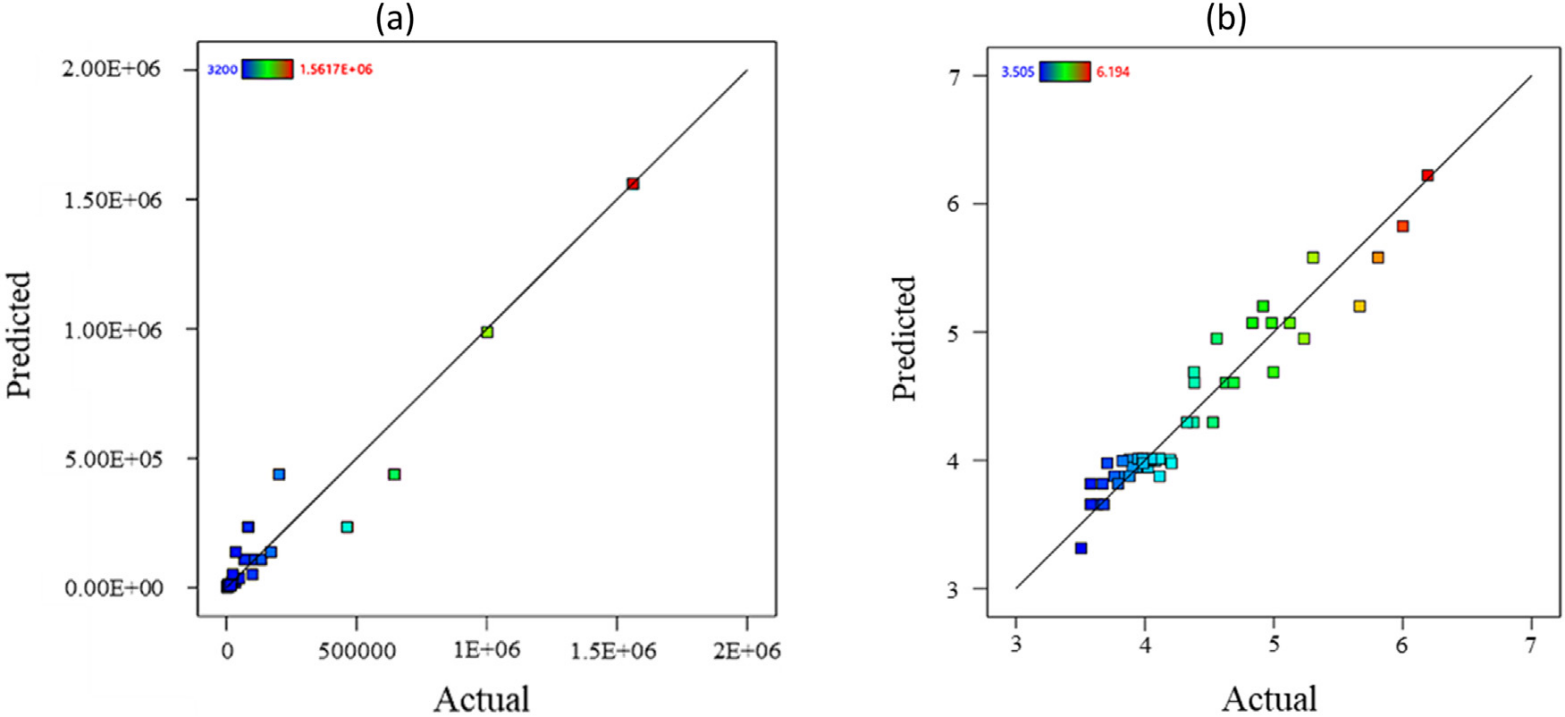
The scatter-band of the actual (experimental) and predicted fatigue lifetime of 16MnCr5 steel alloys, including (a) fatigue lifetime and (b) Log fatigue lifetime.

**Fig. 9:**
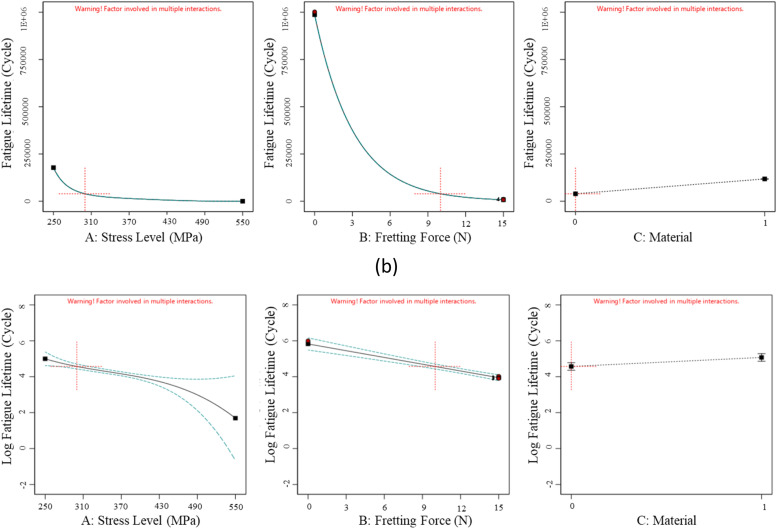
The effect of variables on the fatigue lifetime of 16MnCr5 steel alloys, including (a) fatigue lifetime and (b) Log fatigue lifetime.

**Fig. 10:**
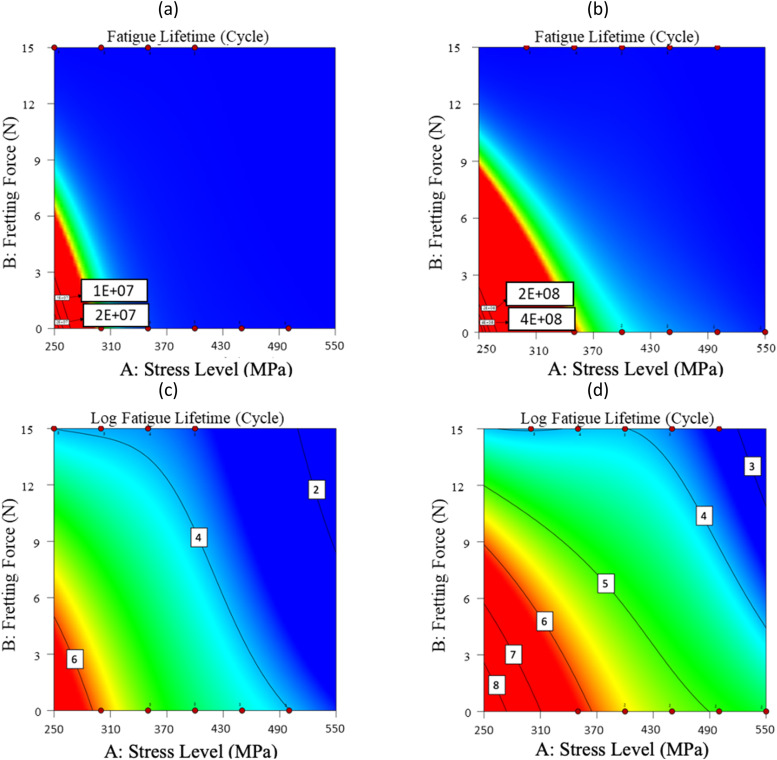
The contour plots of A versus B parameters on (a) the fatigue lifetime for as-received material (C = 0), (b) the fatigue lifetime for coated material (C = 1), (c) the logarithmic fatigue lifetime for as-received material (C = 0), and (d) the logarithmic fatigue lifetime for coated material (C = 1).

Fig. 8 proposes the scatter band for the 16MnCr5 steel alloys for the experimental and predicted data. Figs. 9 and 10 illustrate the impact of each parameter on the fatigue lifetime of 16MnCr5 piston pin steel alloys, both in raw data and logarithmic values. This representation allows for observing the trend behavior of all variables. As anticipated, stress level and fretting force reduced the fatigue lifetime. Conversely, the DLC coating demonstrated a positive effect, benefiting the fatigue properties of the material. For these illustrations, the actual factors (the red plus mark in Fig. 7) were set as follows: stress level (A) was set to 300 MPa, fretting force (B) was set to 10 N, and material (C) was set to as-received. In addition, the second note was the order of influential factors compared to each other. Considering this issue, Fig. 10 presents the contour plots of variable influences on fatigue characteristics.

The analysis of Figs. 9 and 10 concludes that an increase in the stress level and the fretting force reduces fatigue lifetime. The further decrease in the fatigue lifetime upon applying the fretting force is due to the increased surface damage, which accelerates the initiation of fatigue cracks. Moreover, the samples coated with Diamond-Like Carbon (DLC) exhibited a superior fatigue resistance, primarily because of the distinctive characteristics of the coatings. The DLC coating, renowned for its exceptional hardness and low friction coefficient, significantly enhances fatigue resistance. It serves as a protective barrier for the metal beneath, shielding it from environmental factors and diminishing the surface roughness, a potential origin for the crack initiation [16].

Additionally, coating aids in the even distribution of the stress across the surface, thus minimizing the stress concentrations at any given point. The low friction coefficient of DLC coating also reduces wear during fretting, a frequent precursor to premature fatigue failure. The high hardness of DLC coating hinders crack propagation, ensuring that even if a crack does initiate, it is less likely to fail [16]. In summary, the improved fatigue resistance of the DLC-coated samples can be ascribed to the coating capacity to safeguard the surface, evenly distribute stress, minimize wear, obstruct crack propagation, and preserve chemical stability under fretting conditions. This comprehensive protection is vital for components exposed to cyclic loading and high-stress environments, where fatigue failure is a significant concern.

Based on OM investigation (see Fig. 1), the material was carbon steel with a hypereutectoid or ferrite and pearlite structure. A similar microstructure has also been reported in the literature [34]. The microstructure can influence fracture behavior under fatigue loading, which involves repeated stress causing crack initiation and propagation. Smaller grains can enhance fatigue strength and resistance by providing more barriers to crack growth and enhancing the Hall-Petch effect. However, small grains can reduce fatigue strength and resistance by increasing stress concentration and susceptibility to grain boundary cracking. Different phases also exhibit varying mechanical properties and responses to fatigue loading. Intergranular crack growth in 16MnCr5 steel is related to its microstructure. Heat treatments like carburizing, nitriding, and nitrocarburizing enhance mechanical properties by altering surface composition [35].

**Fig. 11:**
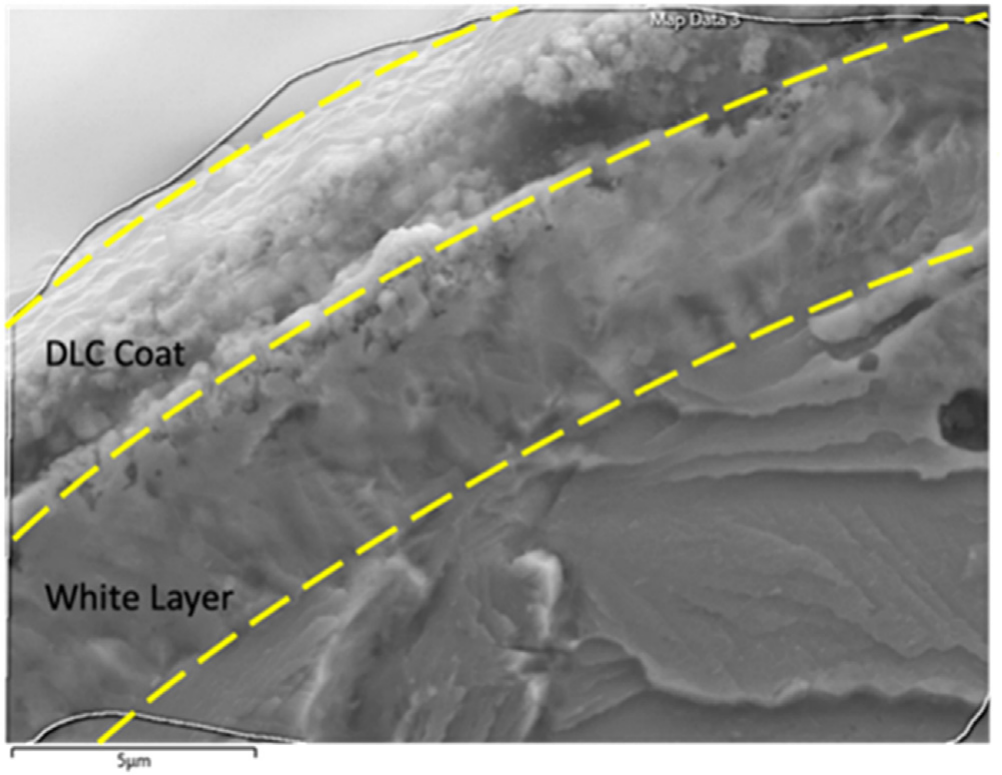
The formation of the DLC and white layers on the DLC-B samples [16].

After duplex plasma nitriding followed by DLC coating, the surface typically consists of multiple layers. The nitrogen-diffusion layer, formed during the plasma nitriding process, enhances surface hardness and wear resistance by diffusing nitrogen into the substrate material. The topmost layer is the DLC coating, which provides a low friction coefficient, high hardness, and excellent wear resistance. Additionally, the white layer, also known as the compound layer, can form during the plasma nitriding process (see Fig. 11). This layer consists mainly of iron nitrides (such as Fe_2–3_ N and Fe_4_ N) and can affect the adhesion of subsequent coatings, like DLC. In some advanced plasma nitriding processes, efforts are made to minimize or eliminate the formation of this white layer to improve the overall performance and adhesion of the DLC coating. This combination results in a surface that is both hard and wear-resistant, making it suitable for various high-performance applications [16].

Additionally, the white layer formed after plasma nitriding represents a vast difference in the elastic module compared to the substance. This can hasten the crack initiation stage and propagation speed, reducing the fatigue lifetime [36]. The ImageJ software measured the thickness of each layer, and the thickness of the DLC coat and the White Layer were 1.956± 0.1478 and 4.4248± 0.5020 micrometers, respectively.

## Limitations

While this study provides insights into the fatigue behavior of 16MnCr5 steel alloy under plain and fretting fatigue conditions after DLC coating, several limitations should be acknowledged:

1. Test Number and Variability: The study was conducted on a limited number of specimens. Naturally, a more significant number of tests could enhance the statistical significance of the results.
2. Environmental Conditions: All experiments were performed at room temperature and under controlled laboratory conditions. However, in real-world applications, such as internal combustion engine components, materials are subjected to varying environmental conditions, such as high-temperature fluctuations and exposure to corrosive elements, which can significantly affect fatigue performance.
3. Fretting Force: The fretting force applied in this study was fixed at 15 N. In practical applications, the fretting force can vary widely depending on the operating conditions. Investigating the effects of different fretting forces could provide a more comprehensive understanding of the fatigue behavior.
4. Stress Levels and Loading Method: The stress levels used in the fatigue tests ranged from 250 to 550 MPa, and the loading method was fully reversed rotary bending. While this range and method cover significant operational stresses, they may only encompass some possible stress scenarios encountered in real-world applications. Further studies could explore a broader range of stress levels and different loading methods.
5. Microstructural Analysis: Although OM and Raman spectroscopy were used for microstructural analysis, other advanced techniques such as TEM or XRD could provide more detailed insights into the microstructural changes and phase transformations occurring during fatigue.

Acknowledging these limitations aims to provide a clear understanding of the scope of the study and constraints, paving the way for future research to build upon these findings.

## Ethics statements

The authors confirmed that the current work does not involve human subjects, animal experiments, or any data collected from social media platforms.

## CRediT authorship contribution statement

**Mobin Dizisaz:** Investigation, Validation, Data curation, Software, Visualization, Data curation. **Mohammad Sadegh Aghareb Parast:** Investigation, Validation, Data curation, Software, Visualization, Writing – original draft, Writing – review & editing. **Mohammad Azadi:** Conceptualization, Methodology, Investigation, Validation, Supervision, Writing – review & editing.

## Declaration of competing interest

The authors declare that they have no known competing financial interests or personal relationships that could have appeared to influence the work reported in this paper.

## Data Availability

Data will be made available on request.
